# HA and HS Changes in Endothelial Inflammatory Activation

**DOI:** 10.3390/biom11060809

**Published:** 2021-05-29

**Authors:** Elena Caravà, Paola Moretto, Ilaria Caon, Arianna Parnigoni, Alberto Passi, Evgenia Karousou, Davide Vigetti, Jessica Canino, Ilaria Canobbio, Manuela Viola

**Affiliations:** 1 Quantix Italia S.r.l., 20121 Milano, Italy; carava@quantixitalia.com; 2 Department of Medicine and Surgery, University of Insubria, 21100 Varese, Italy; paola.moretto@uninsubria.it (P.M.); i.caon@uninsubria.it (I.C.); a.parnigoni@uninsubria.it (A.P.); alberto.passi@uninsubria.it (A.P.); jenny.karousou@uninsubria.it (E.K.); davide.vigetti@uninsubria.it (D.V.); 3 Department of Biology and Biotechnology, University of Pavia, 27100 Pavia, Italy; jessica.canino@iusspavia.it (J.C.); ilaria.canobbio@unipv.it (I.C.)

**Keywords:** heparan sulfate, inflammation, Syndecans

## Abstract

Cardiovascular diseases are a group of disorders caused by the presence of a combination of risk factors, such as tobacco use, unhealthy diet and obesity, physical inactivity, etc., which cause the modification of the composition of the vessel’s matrix and lead to the alteration of blood flow, matched with an inflammation condition. Nevertheless, it is not clear if the inflammation is a permissive condition or a consequent one. In order to investigate the effect of inflammation on the onset of vascular disease, we treated endothelial cells with the cytokine TNF-α that is increased in obese patients and is reported to induce cardiometabolic diseases. The inflammation induced a large change in the extracellular matrix, increasing the pericellular hyaluronan and altering the heparan sulfate Syndecans sets, which seems to be related to layer permeability but does not influence cell proliferation or migration nor induce blood cell recruitment or activation.

## 1. Introduction

Cardiovascular diseases (CVD) are a group of pathologies of the vascular system that are growing in number and that usually present a build-up of fatty deposits inside the arteries (atherosclerosis) and an increased risk of blood clots and can also be associated with damage to arteries in organs such as the brain, heart, kidneys, and eyes. CVD are triggered by several risk factors, ranging from pathologies such as diabetes, hypercholesterolemia, and metabolic syndrome, to unhealthy habits, e.g., inactivity, smoking, and excessive alcohol. Apart from the medical advice that may include changing lifestyle and diet, the only medical treatment that can help decrease the risk is lowering cholesterol levels and controlling blood pressure and coagulation [1,2]. Despite the high numbers of data about CVD, the exact timing and molecular mechanism at the basis of the pathologies are still unclear, even though they are all inflammation related [3,4]. Some authors proposed that some of the risk factors lead to qualitative changes in the endothelium such as changes in permeability and increase in adhesion molecules expression that attract leucocytes (“response of injury” hypothesis) [5,6], which, in turn, cause the inflammatory condition. Moreover, the localized inflammation causes the thickening of the arterial wall due to the increased deposition of extracellular matrix (ECM) and the newly formed ECM traps lipoprotein and inflammatory/growth factors from the circulation within the vessel wall [3].

In obese patients, the endothelium is characterized by dysfunction associated with a condition of vascular low-grade inflammation in which an excess of TNF-α (tumor necrosis factor) is generated either in small vessels or within the perivascular adipose tissue [2]. TNF-α has also an effect on the release of nitric oxide (NO), which is produced to regulate vascular tone, cardiac contractility, and vascular remodeling [7].

The endothelial layer is composed of several components, the major ones including fibrous proteins such as collagen, fibronectin, elastin, laminin, and glycosaminoglycans (GAGs) chains such as hyaluronan (HA) or proteoglycans (PGs). The PGs and GAGs fill the interstitial space of tissues left free from fibrous protein, forming a well-organized network conferring tissue hydration [8] as well as function as co-receptors and therefore regulating their activity and that of growth factors and cytokines [1,9]. Both HA and heparan sulfate (HS) GAGs are pivotal in the maintenance of endothelial function, and their removal causes a loss of barrier properties comparable to an inflammatory condition [10]; moreover, several data pointed out the Syndecan family of HSPG as central in the endothelium behavior [11]. Syndecans are a family of four transmembrane HSPGs that are expressed in a cell-type-specific manner [11]; Syndecan-1 is present during development and in adult endothelium and cancer cells; Syndecan-2 is expressed in liver, mesenchymal tissue, and neuronal cells; Syndecan-3 is a neuronal type; and Syndecan-4 is ubiquitously distributed [12,13]. The complex mechanism of biosynthesis and modification of the HS chains generate a variability in N-sulfation levels along the polysaccharides. EXT1 and EXT2 are the polymerizing enzymes, while glucosaminyl N-deacetylase/N-sulfotransferase (NDST) is the first modification enzyme that starts to work on the growing heparan sulfate (HS) polysaccharide chain. This enzyme defines the sulphation pattern, which will determine the ability of the HS chain to interact with target molecules [14]. Moreover, the NDST1 has the capacity to bind to EXT2, and EXT1 and EXT2 expressions affect the N-sulfation degree, hinting that the overexpression of all the three enzymes happens simultaneously. The subsequent sulfations occur on the growing chains [15]; it is noteworthy that although there is only a single 2-O-sulfotransferase, there are three 6-O-sulfotransferases (6OST1-3) and seven 3-O-sulfotransferases [16], indicating the great importance of the sulfation pattern on the growth factor binding ability of the HS chains [17]. It produces regions rich in N-acetylated residue (GlcA and GlcNAc) called NA domain, regions rich in N-sulfated residue (IdoA and GlcNS derivates) called NS domain, and sequences that contain alternation of NA and NS. The ligands binding mainly depend upon the distribution of these domains [18,19]. The synthetic GAG machinery can be altered by several events, including the changes in PG core proteins expression or UDP-sugars transporters [20].

A preliminary clinical study in patients with resistant arterial hypertension (CVD risk factor) indicates high Syndecan-4 level as a potential marker for endothelial dysfunction [21]; moreover, the same HSPG has also been shown to regulate focal adhesions junctions, demonstrating the properties of a mechano-transducer effector [22], and importantly, its fragments generated following thrombin cleavage can modulate changes in endothelial barrier resistance [23]. Since one of the major events in atherosclerosis onset is the accumulation in the sub-endothelium of lipids driven by the lipoprotein LDL, the passage of such particles through the endothelial barrier is a critical step. Nevertheless, despite the many studies underscoring the relevance of plasma lipoproteins and the effects of lipids and cholesterol on the cell behavior and extracellular matrix architecture of the tunica intima [3], the data about the events causing the LDL transcytosis and accumulations are still scant. Links regarding Syndecan-4 metabolism/levels and the endothelial barrier system are not yet known.

Following the data reported, we made endothelial cells undergo inflammatory conditions using the TNF-α cytokine and investigated the effect in the main ECM components, i.e., HA and Syndecans, evaluating their effect on cell behavior, with regards to the pro-atherogenic aspect of membrane permeability.

## 2. Materials and Methods

### 2.1. Material

HUVEC, human umbilical vein endothelial cells (Gibco, Waltham, MA, USA); M200 culture medium (Gibco, Waltham, MA, USA); DMEM High Glucose w/o Sodium Pyruvate w/ L-Glutamine (Euro Clone, Pero, Italy); TNF-α (Sino Biological Inc., Wayne, NJ, USA); Protease Inhibitor Cocktail (Sigma-Aldrich, St. Louis, MO, USA); Hyaluronate Lyase from Streptomyces hyalurolyticus (Sigma-Aldrich, St. Louis, MO, USA); Transwell system with filter of 0.4 µm pore size, 6.5 mm diameter (Corning, New York, NY, USA); FITC labelled dextran (Mw~250,000, Sigma-Aldrich, St. Louis, MO, USA); heparinases I–II–III (form F. heparinum, Seikagaku, Tokyo, Japan); 2-Aminoacridone, AMAC-(Sigma-Aldrich, St. Louis, MO, USA); Chondroitinase ABC (from Proteus vulgaris, Seikagaku, Tokyo, Japan); 3-hydroxybiphenol (Fluka, Buchs, Switzerland); D-Glucuronic acid (Sigma-Aldrich, St. Louis, MO, USA); prostaglandin E1 and indomethacin (Sigma); all chemicals were purchased by Sigma-Aldrich (St. Louis, MO, USA).

### 2.2. Methods

#### 2.2.1. Cell Cultures

Human umbilical vein endothelial cells (HUVEC) obtained from Gibco (Waltham, MA, USA), were grown for 4–8 passages in M200 culture medium (Gibco, Waltham, MA, USA) supplemented with 2% fetal bovine serum (FBS). The cultures were maintained in an atmosphere of humidified 95% air, 5% CO_2_, at 37 °C. Twenty-four hours before treatments, subconfluent HUVEC were cultured in DMEM with 0.5% FBS. The medium was then changed to M200 with 0.1 μg/mL of TNF-α (Sino Biological Inc., Wayne, NJ, USA) and incubated for 24 or 48 h.

#### 2.2.2. Quantitative RT-PCR

Total RNA samples were extracted from untreated or treated cells with an Absolutely RNA Microprep Kit (Agilent Technologies, Santa Clara, CA, USA). cDNA was generated by using the High-Capacity cDNA synthesis kit (Applied Biosystems, Foster City, CA, USA) and amplified on an Abi Prism 7000 instrument (Applied Biosystems, Foster City, CA, USA) using the Taqman Universal PCR Master Mix (Applied Biosystems). The following human TaqMan gene expression assays were used: HAS2 (Hs00193435_m1), HAS3 (Hs00193436_m1), NOS1 (Hs00167223_m1), NOS2 (Hs01075529_m1), NOS3 (Hs01574659_m1), SYND1 (Hs00174579_m1), SYND2 (Hs00299807_m1), SYND3 (Hs00206320_m1), SYND4 (Hs00161617_m1), NDST1 (Hs00155454_m1), EXT1 (Hs00609162_m1), EXT2 (Hs00181158_m1), and β-actin (Hs99999903_m1) as the reference gene. The relative quantification of gene expression levels was determined by comparing 2^-ΔΔCt [3,24] using non-treated cells, or the expression of HAS2 and Syndecan-1 as a normalizers.

#### 2.2.3. Western Blotting

A RIPA buffer (50 mM Tris (pH 7.4), 150 mM NaCl, 1% TRITON X-100, 0.5% sodium deoxycholate, 0.1% SDS) containing Protease Inhibitor Cocktail (Sigma-Aldrich, St. Louis, MO, USA) was used to prepare cell lysates. Proteins were quantified, separated in a 12% SDS polyacrylamide gel electrophoresis, and transferred to nitrocellulose membrane. After incubation in blocking solution, 5% BSA in TBS-T (Tris-Buffered Saline: 0,02 M Tris, 0,136 M NaCl, 0,001 % Tween-20, pH 7.6), the membrane was incubated overnight with a primary antibody at 4 °C. Antibodies used were rabbit polyclonal antibody against Syndecan4 (ABT157, Merck Millipore, Burlington, VT, USA) dilution 1:250, and goat polyclonal antibody against β-actin (#J1805, Santa Cruz Biotechnology, Dallas, TX, USA), dilution 1:1000. The membrane was washed with TBS-T and incubated for 1 h with the secondary antibody. Band visualization was carried out by the chemiluminescence system LiteAblot TURBO (Euro Clone, Pero, Italy). The relative intensities of the protein bands were analyzed with ImageJ software. β-actin levels were used as controls for protein loading.

#### 2.2.4. Cell Transfection

HUVEC were transfected with siRNA against syndecans4 (S12639, Ambion, Carlsbad, CA, USA) using a nucleofector apparatus (Amaxa, Basel, Switzerland) and the Amaxa HUVEC Nucleofector kit (Lonza, Basel, Switzerland) following the manufacturer’s instructions. In total, 5 × 100,000 cells were resuspended in 100 µL HUVEC Nucleofector solution and transfected with 40 nM siRNA against syndecans4 and Silencer Negative Control siRNA #1 (AM4611, Ambion, Carlsbad, CA, USA). Syndecans4 silencing efficiency was determined by qRT-PCR. Silenced cells were treated 24 h after the transfection.

#### 2.2.5. Cell Viability Assay

HUVEC metabolic activity was evaluated with the MTT assay. Cells were plated at a density of 6 × 1000 cells/well in a 96-well plate. After 16–18 h, HUVEC were treated with 0.1 µg/mL of TNF-α. After 4, 16, 24, or 48 h, the cells were washed with PBS, and MTT solution (50 μL of 5 mg/mL) was added to each well for 4 h at 37 °C. Subsequently, the medium was removed, and DMSO (Sigma-Aldrich, St. Louis, MO, USA) was added (200 µL/well) to solubilize the formazan crystals. Optical density was measured at 570 nm with the Tecan microplate reader (Thermo Scientific, Waltham, MA, USA).

#### 2.2.6. Migration Assay

HUVECs were cultured until confluence in 6-well plates and serum-deprived (0.2% FBS) for 16–18 h. Three scratches per well were done with a 20 μL sterile pipette tip. Cells were washed to remove debris and incubated in fresh M200 with or without TNF-α. Images from three different scratch areas in each culture well were obtained using Olympus (Hamburg, Germany) IX51 microscope after 2, 4, 6, and 8 h.

#### 2.2.7. Exclusion Assay

HUVEC pericellular coat was visualized and measured by using a particle exclusion assay. In total, 6 × 1000 cells/well were seeded in 12-well plate and treated with TNF-α or PBS as control. After 24 h, 500 μL of a suspension of formaldehyde-fixed erythrocytes (15 × 1,000,000 erythrocytes/mL) was added to the wells and allowed to settle for 20 min at 37 °C. Images of the pericellular coat were obtained using phase contrast microscope Olympus IX51. The presence of HA on the pericellular coat was evaluated treating the cultures with 2 U/mL of Hyaluronate Lyase for 1 h at 37 °C before visualization with the particle exclusion assay. Representative cells were photographed at a magnification of ×40; the control experiment was performed with heat inactivated Hyaluronate lyase. ImageJ software was used to quantify the area delimited by red blood cells and the area delimited by the cell membrane to give a coat-to-cell ratio [25].

#### 2.2.8. Permeability Assay

FITC-labelled dextran was used as the representative of hydrophilic molecules to measure the permeability of endothelial cell monolayer [26,27]. HUVEC were plated in the upper part of a Transwell filter with 0.4 µm pore size at a density of 8 × 1000 cells per well until the formation of a tight monolayer was checked with the microscope. Cells were treated with TNF-α (0.1 µg/mL), and FITC-dextran was added to the top chamber of the Transwell in a final concentration of 1 mg/mL. The culture medium in the upper and in the lower chamber was collected 24 h post-treatment, and fluorescence was measured by fluorimeter (Tecan, Thermo Scientific, Waltham, MA, USA) with an excitation wavelength of 490 nm and an emission wavelength of 520 nm. To evaluate the FITC-dextran passage through the cells monolayer, we calculated the percentage of lower over total fluorescence.

#### 2.2.9. Glycosaminoglycans Purification and Quantification

Glycosaminoglycan from the culture medium or cell membrane was extracted using the protocol described in Viola et al. [28]. Briefly, after sample stimulation, conditioned media were collected as well as trypsin supernatants after cells harvesting (membrane GAGs). Samples were subjected to digestion with proteinase K (20 U/mL, Finnzymes, Espoo, Finland) and precipitation with ethanol (9:1 / ethanol:water).

HE/HS Δ-disaccharides were obtained digesting the pellet with a mix of heparinases I–II–III (form F. heparinum, Seikagaku, Tokyo, Japan) 0.5 U/mL each and then derivatized with AMAC (Sigma-Aldrich St. Louis, MO, USA). HE/HS Δ-disaccharides were analyzed and quantified by HPLC with respect to specific standards.

Intact HS GAGs were purified digesting the pellet with 0.1 U/mL U of Chondroitinase ABC for 5 h at 37 °C.

HS GAGs amount was calculated by means of the uronic acid content, using the van den Hoogen et al. method [29]. Briefly, 40 µL of the HS sample and 200 µL of concentrated sulfuric acid (80% *w*/*w*) were added in a 96-well plate. The plate was incubated for 1 h at 80 °C and, after cooling to room temperature, the background absorbance of samples was measured at 540 nm on a microplate reader (Tecan, Thermo Scientific, Waltham, MA, USA). Then, 40 µL of 3-hydroxybiphenol solution (100 µL of 100 mg/mL 3-hydroxybiphenol in DMSO mixed with 4.9 mL 80% (*v*/*v*) sulfuric acid) was added. After an overnight incubation, the absorbance was read again at 540 nm. D-Glucuronic acid (Sigma-Aldrich St. Louis, MO, USA) was used for a standard curve.

#### 2.2.10. Data Analysis

Data are presented as mean ± S.E.M. Statistical significance was determined using Student’s *t* test. Statistical significances were *p* < 0.05 for *, *p* < 0.01 for **, and *p* < 0.001 for ***.

## 3. Results

In the development of atherosclerosis, the endothelial dysfunction is one of the beginning steps or a permissive status of the endothelial layer for the onset of the pathology. Treatment of HUVEC cells with TNF-α can mimic the systemic inflammatory status of the endothelium [30,31].

**Figure 1:**
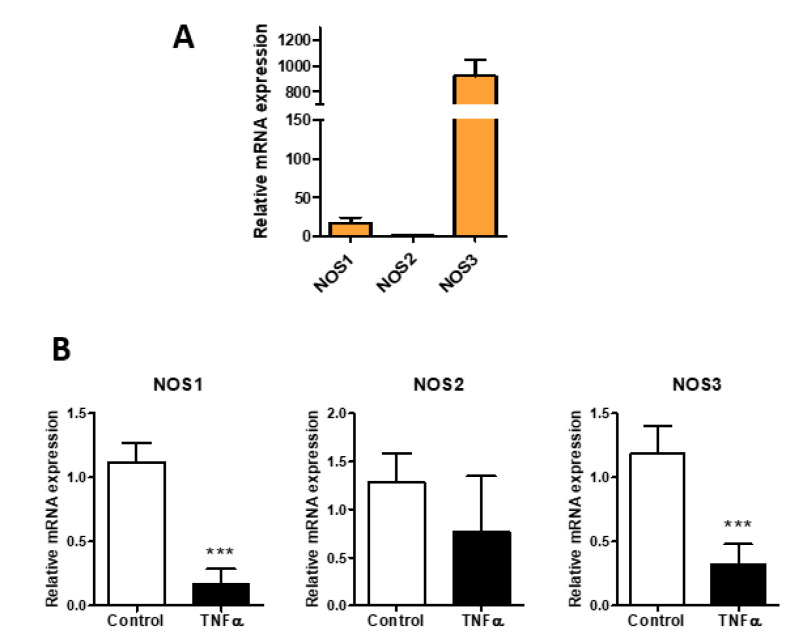
Effect of TNF-α on NO synthetic enzymes in HUVEC. (**A**) relative expression of NOSs (neuronal NOS1, inducible NOS2, and endothelial NOS3) in HUVEC. (**B**) NOSs expression in HUVEC untreated (control) and treated with TNF-α (0.1 µg/mL) for 24 h. Data are mean ± S.E.M. of three independent experiment, *** *p* < 0.001.

In order to confirm the inflammatory condition of HUVEC cells, we analyzed nitric oxide synthases (NOSs) expression. Nitric oxide (NO) is important to maintain normal vascular functions and endothelial integrity. As expected, the endothelial isoform NOS3 was the most expressed form in HUVEC (Figure 1A), and the expression levels of NOS3 and NOS1 were significantly decreased after TNF-α stimulation, while NOS2 showed a non-significant tendency to decrease (Figure 1B). These data agree with the literature in which in vitro studies confirm the defect in the NO production in isolated atherosclerotic blood vessels [7,32].

Migration recorded through 8 h and vitality at 24 and 48 h were not affected by the cytokine (Supplementary Figure S1).

The extracellular matrix expressed by endothelial cells is commonly referred to as glycocalyx and has an important role in controlling shear stress from laminar flow through mechano-transduction mechanisms [14] and inflammation, thus controlling cell adhesion, motility, and proliferation [15,16,17].

**Figure 2:**
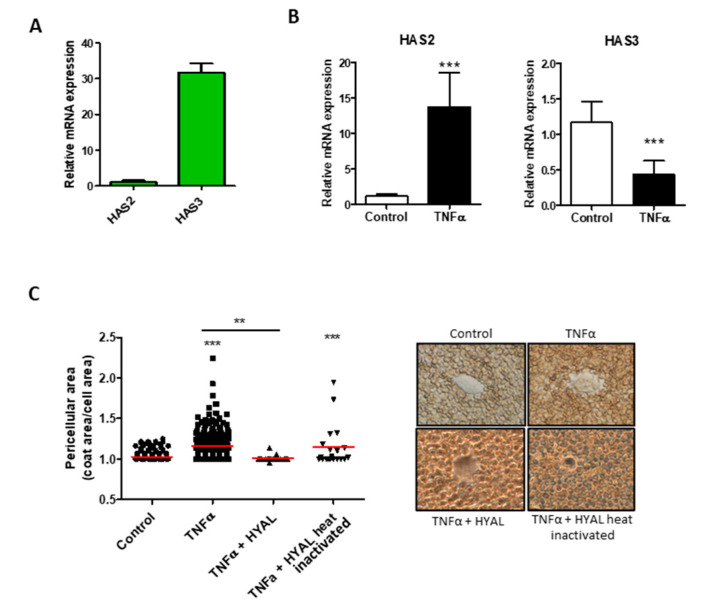
Effect of TNF-α on Hyaluronan synthesis in HUVEC. (**A**) HASs expression profile in HUVEC. The reference gene used for normalization was β-actin and the normalizer HAS2 expression. (**B**) Relative expression of HAS2 and HAS3 after TNF-α stimulation (24 h). The reference gene used for normalization was β-actin and the normalizer untreated samples. Data are mean ± S.E.M. of four independent experiments, *** *p* < 0.001. (**C**). Particle exclusion assay performed on HUVEC untreated (control) and under TNF-α stimulation for 24 h. To clarify the HA composition of the pericellular matrix, we digested HA with 2 U/mL of Hyaluronate Lyase from Streptomyces hyalurolyticus (HYAL) before the addition of erythrocytes. Original magnification 40×. Values represent the measure of the single cell pericellular area, and the red bars are the mean of three independent experiments, *** *p* < 0.001 and ** *p* < 0.01.

The HA production has also an important role in the maintenance of cell homeostasis and in activation of different signal transduction pathways [33]. In HUVEC cells, HAS3 mRNA was the most abundant (Figure 2A), whereas HAS1 messenger was not detected (data not shown). Interestingly, after TNF-α stimulation, HAS2 increased expression while HAS3 was decreased (Figure 2B). In order to evaluate the glycocalyx of the HUVEC, we quantified the glycosaminoglycans from the membrane and from the medium with no significant differences (Supplementary Figure S3). The pericellular coat surrounding the endothelial cells was measured and showed a significant increase after TNF-α stimulation, which was mainly constituted of HA as demonstrated by enzymatic digestion (Figure 2C).

**Figure 3:**
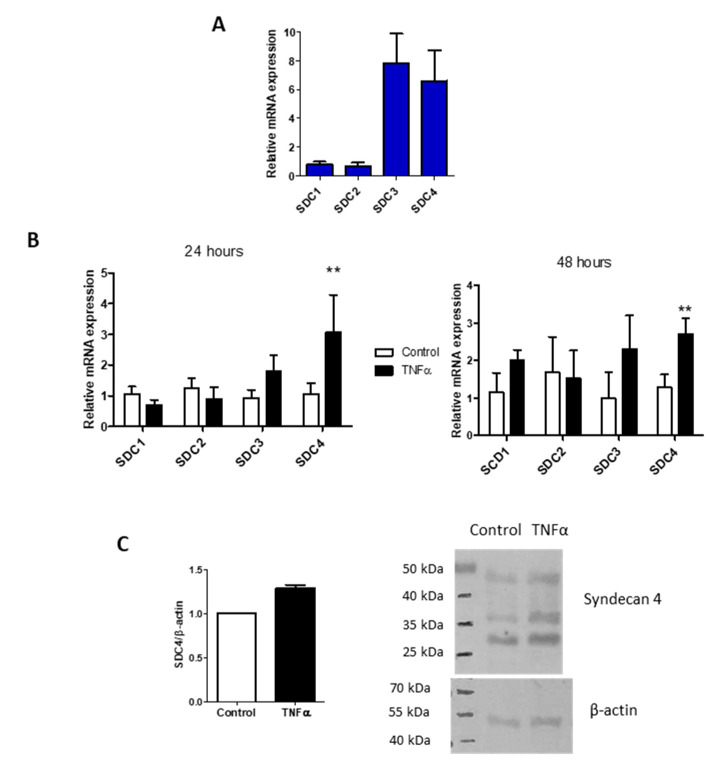
TNF-α influence on Syndecans expression. (**A**) Syndecans expression profile in HUVEC. The reference gene used for normalization was β-actin and the normalizer the Syndecan-1 expression level. (**B**) Syndecans isoforms expressions in HUVEC control and after 24- and 48-h of TNF-α stimulation. The reference gene used for normalization was β-actin and the normalizer untreated samples. Values represent mean ± S.E.M. (n = 3), ** *p* < 0.01. (**C**) Western blot analysis of Syndecan-4 (SDC4) protein in HUVEC control and treated 24 h with TNF-α. Bar chart represents normalized mean ± S.E.M. of two independent experiments and the figure is a representative SDS-PAGE.

Due to the lining of the vessels, ECM is also important in recruitment and activation of immune cells and of platelets from the blood [34,35,36]. Among all the HSPGs, Syndecans are a family of four transmembrane proteoglycans acting as co-receptors interacting with different molecules including growth factors, matrix components, and cytokines that are present in glycocalyx [11]. In HUVEC, the main Syndecans expressed are the -3 and -4 isoforms (Figure 3A), but only Syndecan-4 increased during TNF-α stimulation from 24 up to 48 h (Figure 3B). The core protein of the proteoglycan was evaluated in the cell extraction and shown in Western blot and turned out to be increased, even if not significantly (Figure 3C). The Western blot shows three different bands positive to antibody recognition. The three different bands at around 27, 37, and 45 kDa can be the proteoglycan with different GAG chains [37], Syndecan-4 bound to growth factor or matrikines, and/or its homo- or hetero-oligomerization forms [38].

As reported, the NDST1 has the capacity to bind to EXT2, and EXT1 and EXT2 expressions affect the N-sulfation degree, suggesting that the overexpression of all the three enzymes happens simultaneously, as shown in Supplementary Figure S3.

The GAG moiety of the HUVEC HSPGs was analyzed by enzymatic digestion followed by HPLC analysis, and the disaccharide percentages are reported in Table 1. The main drastic difference seems related to the increment of N-sulfation on glucosamine residue. The higher amount of N-sulfation correlates well with the increment of expression of the enzyme NDST1, heparan sulfate N-deacetylase/N-sulfotransferase 1 (Supplementary Figure S3), that catalyzes both the N-deacetylation and the N-sulfation of glucosamine.

The major event in atherosclerosis onset is the accumulation in the sub-endothelium of lipids driven by the lipoprotein LDL [3]; nevertheless, the data about the events causing the LDL particle transcytosis and accumulations within the tunica intima are still scant.

**Figure 4:**
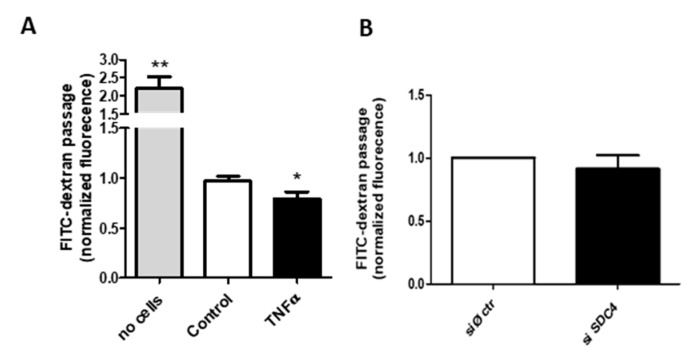
Transwell permeability assay. (**A**) FITC-dextran flow through HUVEC monolayer. Confluent HUVEC cells in the upper chamber of a transwell system +/− TNF-α were added with 1 mg/mL of dextran conjugated with FITC. After 24 h, the medium of the lower chamber was collected and FITC fluorescence was measured, * *p* < 0.05 and ** *p* < 0.01. (**B**). FITC-dextran flow through siRNA control (siØ ctr) or siRNA against SDC4 HUVEC monolayers; data are mean ± S.E.M. and n = 3.

To test whether the endothelial permeability is altered under the inflammatory condition, we incubated a continued layer of HUVEC in a transwell system with FITC-dextran using a permeable membrane with a cut-off unable to let the cells pass. As reported in Figure 4A, the presence of the HUVEC layer (control) blocks the free passage of the fluorescent dextran, and the same cell under the inflammatory condition of TNF-α increases the blocking by a significant, even if small, amount.

Since Syndecan-4 exerts various effects on the endothelial glycocalyx, with particular regard to TNF-α induced endothelial modifications [40], and has a pivotal role in the dynamics of focal adhesion [41] and in the formation of networks at gap junctions [42], we investigated the HUVEC permeability of SDC4-silenced cells (Figure 4B) (silencing efficiency 80%, data not shown). The abrogation of the proteoglycan does not alter the dextran passage, even considering the high silencing levels, thus indicating a complex metabolism and turnover for the proteoglycan as well as multiple control levels for the layer permeability.

## 4. Discussion

The atherosclerotic process in which we are interested is a combination of several factors, among which high cholesterol levels, driven by lipoprotein LDL, vasculature inflammation, and oxidative processes are the main components, deeply studied but still not easily correlated [1,3,43]. In particular, it is not yet clear whether the vessel inflammatory condition can influence the LDL passage to the subendothelial space and alter the activation of blood components through the interaction with the glycocalyx. In the in vitro model of inflammation and atherosclerosis using endothelial cells [44] and smooth muscle cells [3], we highlighted the role of the GAG HA in recruiting monocytes/macrophages from blood, promoting their adhesion to the endothelial layer, and altering the ECM composition and thickness of the intima layer.

We determine to use the pro-inflammatory cytokine TNF-α, since it is well known that is produced in the visceral fat of obese and overweight patients that are prone to CVD. Moreover, this chronic low-grade vascular inflammation is hypothesized to be the stimulus by which, within vasculature, reactive oxygen species (ROS) are generated through NAD(P)H oxidase activation and other sources, which in turn reduces NO availability, causing the local endothelial dysfunctions [2]. Therefore, TNF-α can contribute to vascular changes, favouring the development and acceleration of the atherothrombotic process in the clinical condition [2]. In this view, we used the pro-inflammatory cytokine TNF-α treatment on HUVEC cells as a model of vasculature inflammation, investigating the glycocalyx modifications and their effect of the endothelial barrier.

The effects of TNF-α on the NO production shown in Figure 1 confirm the effectiveness of the treatment, and even if the data are only the expression of the synthetic enzymes, this evidence is related to the NO levels [45]. The action of TNF-α on the HA synthesis is reported in Figure 2 and confirms the data previously found with a different cytokine, the IL-1β. Briefly, the synthesis of HA is increased by means of the overexpression of the membrane synthases HAS2, and the GAG is mainly localized around the cells as a pericellular coat that increases the distance between the cells. Among the synthetic HA enzymes, HAS2 is known to be the main producer of the polymer [3]. The HAS3 enzyme decrease in this phase is not yet well understood and deserves other investigations in order to unravel the exact role of the enzyme in the membrane. During the early stage of atherosclerotic lesion formation in Apolipoprotein E (Apoe)-deficient mice, the HAS3 expression is increased and controlled in vascular smooth muscle cells by the cytokine IL-1β [46], and even if data on endothelial cells are unavailable, there are clear indications that HAS3 might be a promising therapeutic target in atherosclerosis.

It is noteworthy that this pericellular coat can increase the adhesion and recruitment of the circulating monocytes [44] and protect cells from apoptotic events [47]. Since HA can also exert its effect on cell proliferation and migration depending on its dimensions [48,49,50] and organized sovramolecular architecture by soluble factors (e.g., TSG-6) [51], the mere synthesis of the polymer is not predictive of its effect. In our model, in fact, the accumulation of HA in the pericellular coat does not alter cell proliferation and migration as reported in Supplementary Figures S1 and S2, which was in line with the barrier role of the endothelial cells, but nevertheless can favour the adhesion and transmigration of blood cells to the sub-endothelium.

Inflammation is the cause of multiple effects on the endothelium, including changing the glycocalyx composition [10,19,52]. The heparan sulfate proteoglycan belonging to the Syndecans family is largely involved in the onset of different CVD in vasculature, and in particular is reported to be increased in patients with resistant hypertension [21], while syndecan-3/-4 ectodomain fragments, produced by several stimuli, including heparinase or thrombin, decrease endothelial cell–cell adhesive barrier integrity [23] and are involved in the cell-extracellular matrix and cell–cell adhesion mechanisms [22]. HSPGs in endothelial cells include glypicans, located on the cell membrane, anchored by glycosylphosphatidylinositol (GPI), functioning mainly as modulators of growth factor signaling [53], but they seem more critically involved in developmental morphogenesis and positively correlate with the onset of certain types of cancers [54] and less with vascular inflammation.

The TNF-α treatment on HUVEC cells selectively increases the expression of Syndecan-4, and the expression remains high after the incubation for up to 48 h (Figure 3); the increase of syndecan-4 is also evident at the protein level, and is measurable with Western blot, even if the results are not significant. This finding can be either due to the low level of expression of the PG in the cells that do not consent to the change to be significant in the assay sensibility range, or to the turnover rate of Syndecan-4, which is formed by the protein core and the GAG moiety. In our data, in fact, we investigated the composition of the heparan sulfate chains of the membrane-bound PGs as well as those released in the medium and evidenced a difference in the percentage of various disaccharides. The different sulfation of the disaccharides can lead to a modification in the sulfation pattern of the GAG chains, and this can be the real important event for the Syndecan-4 related effects, while the protein core can be maintained at the same amount but continuously replaced.

The total amount of uronic containing GAGs remains invariable (Supplementary Figure S4), indicating a balance between the synthesis of the polymer chains that use the same UDP-sugar precursors as we underlined in a previous paper, involving, in particular HA and HS [20], but a control on the number and dimension of the various chains was impossible. As shown in Table 1 the main changes in disaccharides involve the NS and 6 sulfation. Unfortunately, the small size of the samples and high biological variability among them make it impossible to have statistically significant data, but the trend is very sharp. Together with the changes in the protein core of Syndecan-4 and in the NS sulfation, the synthetic enzymes EXT1 and EXT2, responsible for the polymerization, and NDST1 (N-deacetylase/N-sulfotransferase) are also increased in the TNF-α treated samples (Supplementary Figure S3). The sulfation pattern of the HS chains is frequently found altered in the inflammatory condition of tumours, as reported by several papers that indicate specific HS sulfotransferase as critical for the survival and invasiveness of those cells, for example, the 3-O-sulfotransferase [55] or the 2-O-sulfotransferase [56]. Moreover, the 6-O-sulfation of HS highly influences the polysaccharide structural diversity and is critically involved in the binding of many proteins, in particular growth factors [17].

The NDST1 enzyme is the component that catalyses both the N-deacetylation and the N-sulfation of glucosamine (GlcNAc) residue in the heparan sulfate. This enzyme modifies the GlcNAc-GlcA disaccharide-repeating sugar backbone to make N-sulfated heparosan, a prerequisite substrate for later modifications in heparin biosynthesis, such as 6-O-sulfation [15]. Therefore, the increase in the N-sulfation along the chain consequently also increases the sulfation in the C6, as summarized in Table 1. This modification seems more important in the chains bound to the membrane, i.e., carried on the proteoglycan core. For what concerns the CVD, it was reported that the protein PCSK9, an important drug target because of its crucial role in lipid metabolism, can interact with HS in N-sulfation rich domain [57], eventually linking the high cholesterol load with the vasculature inflammation at the onset of the pathology.

The mechanism of endothelial dysfunction involves the alteration of the layer permeability demonstrated in Figure 4, which is dependent upon the overexpression of Syndecan-4, while the silencing of the protein core of the PG does not change the barrier function of the membrane. The increase in Syndecan-4 after treatment with TNF-α can cause a rearrangement in the adhesion asset of the endothelial layer due to its involvement in the dynamics of focal adhesion and the formation of networks at gap junctions. The different integration of the cells with each other and with the basement membrane can be the molecular mechanism leading to lower permeability of the endothelial layer. Nevertheless, this mechanism needs further investigations to be clarified, in particular regarding the interaction with other membrane components.

## 5. Conclusions

This research work aims to close small gaps in the sequence events on the onset of vascular lesion at the basis of various cardiovascular diseases, such as atherosclerosis.

The hypothesis we followed is the establishment of a first inflammatory condition due to physical and environmental state (diet and sport exercise habits, health conditions, etc.), and the results we obtained seem to positively correlate with the onset of a pathological state: (i) alteration of the endothelial barrier properties (i.e., membrane permeability); (ii) increase of HA in the pericellular coat and therefore of the monocyte recruitment possibility from blood; (iii) alteration of the sulfation pattern of membrane-bound HS which can cause modifications of the endothelium response to growth factor and cytokines, as well as of the lipid metabolism through the association HS/PCSK9/LDL-receptor; (iv) HAS3 enzymes abundantly decrease in these conditions without affecting the HA amount, which suggests a different role in the cell behaviour.

Concluding, we can assess that inflammation is the leading event of CVD and that it is of pivotal importance to understand if the inflammatory HS carries specific sequences connected to the various events and unravel the role of HAS3 in endothelial cells.

## Figures and Tables

**Table 1:** HPLC analysis of the main HS/HE disaccharides. GAGs were isolated from plasma membrane and from culture medium of HUVEC control and TNF-α treated (24 h). To obtain HS/HE disaccharides, we digested GAGs with heparinases. After AMAC derivatization, the disaccharides were analyzed by means of HPLC. Data are expressed as % area of each HS disaccharide/ % area total. The N-sulfation in bold (NS) is catalyzed by NDST1. Values are mean ± SD of three independent experiments ** *p* < 0.01. UA: uronic acid; GlcNAc: N-acetyl; GlcNS: N-sulphonyl glucosamine: S: sulphate group [39].

	GAG medium		GAG membrane	
	Control	TNF-α	Control	TNF-α
ΔUA-2S-β[1→4]-Glc**NS**-6S	0.7 ± 1.3	0.4 ± 0.7	4.9 ± 6.9	5.5 ± 7.8
ΔUA-β[1→4]-Glc**NS**-6S	1.2 ± 1.0	2.9 ± 0	0	91 ± 6 **
ΔUA-2S-β[1→4]-Glc**NS**	0.4 ± 0.7	2.5 ± 4.3	0.9 ± 1.3	0.7 ± 0.5
ΔUA-β[1→4]-Glc**NS**	3.8 ± 5.0	34 ± 21	34 ± 23	0.2 ± 0.2
ΔUA-β[1→4]-GlcNAc-6S	66 ± 2	48 ± 36	55 ± 21	0
ΔUA-β[1→4]-GlcNAc	50 ± 39	13 ± 19	5.3 ± 6.6	2.3 ± 0.7

## Supplementary Materials

The following are available online at https://www.mdpi.com/article/10.3390/biom11060809/s1, Figure S1: MTT vitality assay. Figure S2: Wound healing assay of HUVEC. Figure S3: Expression levels of Syndecan chains biosynthetic enzymes. Figure S4: GAGs quantification.
