# Supplementary material for: HA and HS Changes in Endothelial Inflammatory Activation
Source: Biomolecules. 2021 May 29;11(6):809. doi: 10.3390/biom11060809 (PMC8229641; doi:10.3390/biom11060809)
Supplement: Supplementary file 1 [file biomolecules-11-00809-s001.zip › Supplemental_figures.pptx]

## Slide 1
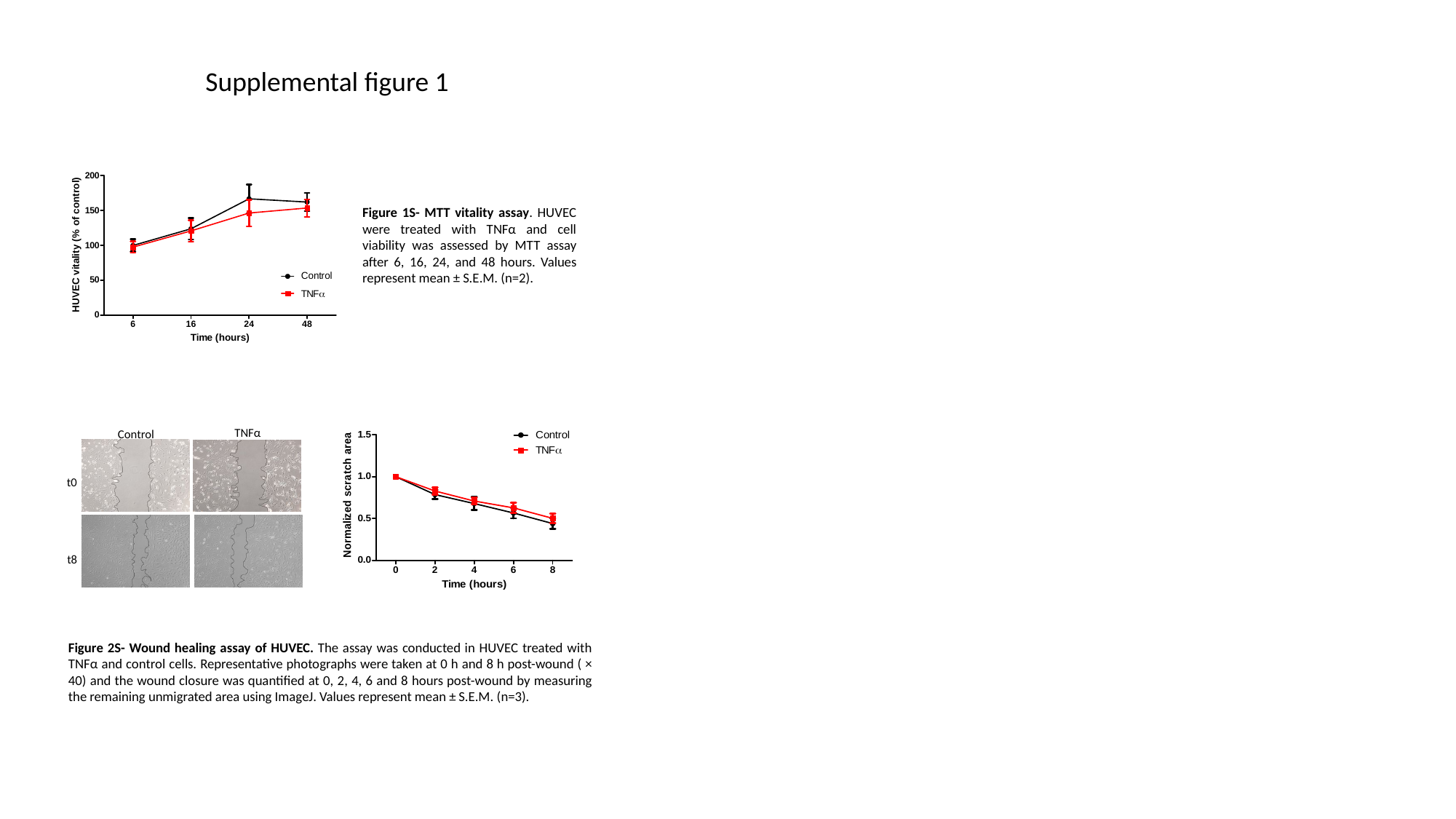

Supplemental figure 1
Figure 1S- MTT vitality assay. HUVEC were treated with TNFα and cell viability was assessed by MTT assay after 6, 16, 24, and 48 hours. Values represent mean ± S.E.M. (n=2).
TNFα
Control
t0
t8
Figure 2S- Wound healing assay of HUVEC. The assay was conducted in HUVEC treated with TNFα and control cells. Representative photographs were taken at 0 h and 8 h post-wound ( × 40) and the wound closure was quantified at 0, 2, 4, 6 and 8 hours post-wound by measuring the remaining unmigrated area using ImageJ. Values represent mean ± S.E.M. (n=3).

## Slide 2
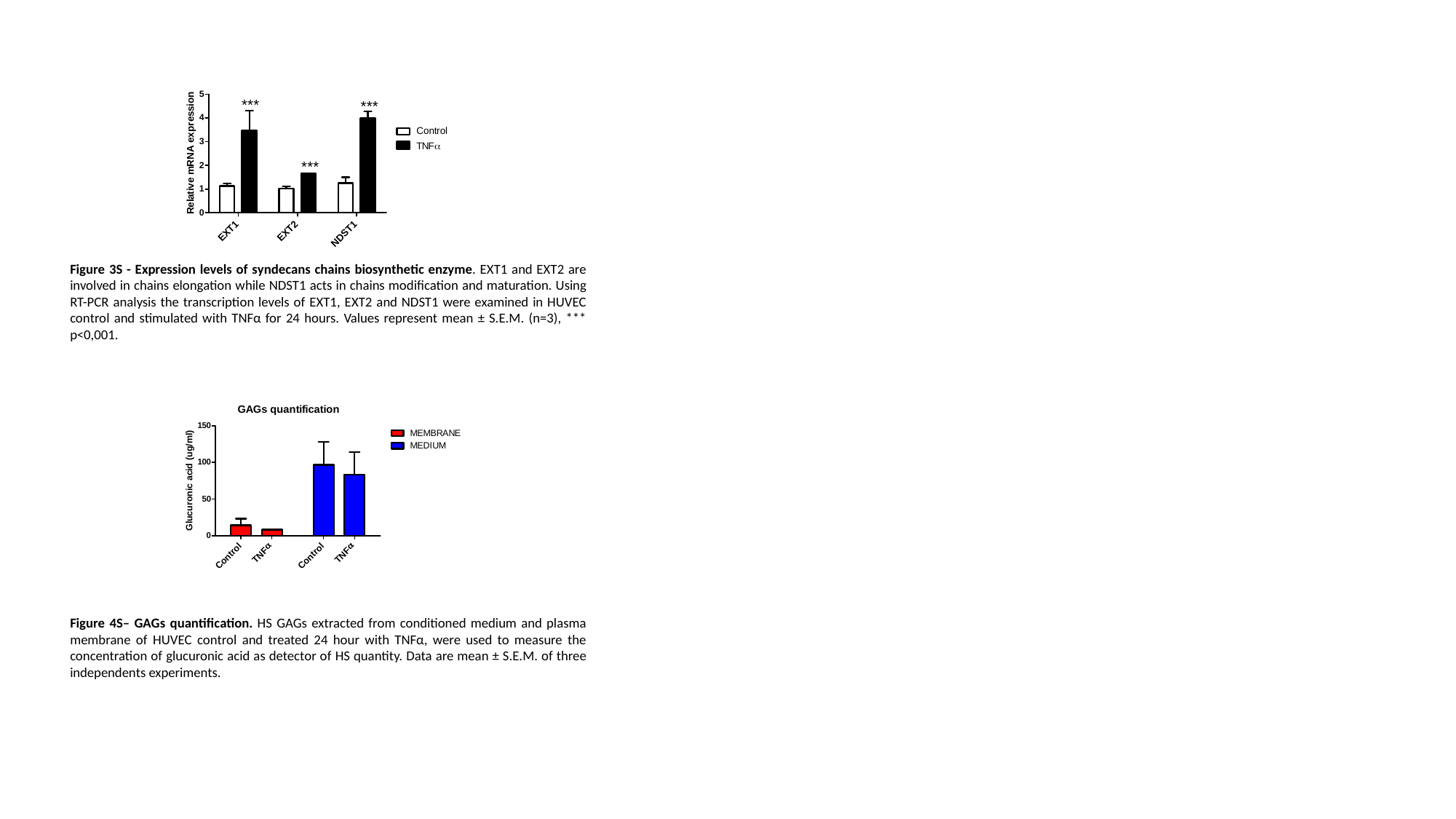

Figure 3S - Expression levels of syndecans chains biosynthetic enzyme. EXT1 and EXT2 are involved in chains elongation while NDST1 acts in chains modification and maturation. Using RT-PCR analysis the transcription levels of EXT1, EXT2 and NDST1 were examined in HUVEC control and stimulated with TNFα for 24 hours. Values represent mean ± S.E.M. (n=3), *** p<0,001.
Figure 4S– GAGs quantification. HS GAGs extracted from conditioned medium and plasma membrane of HUVEC control and treated 24 hour with TNFα, were used to measure the concentration of glucuronic acid as detector of HS quantity. Data are mean ± S.E.M. of three independents experiments.
